# Virtual Reality-Based Medical Education in Ophthalmology: A Scoping Review

**DOI:** 10.7759/cureus.83845

**Published:** 2025-05-10

**Authors:** Briana L Krutsinger, Julia C Moore, James D Colquitt

**Affiliations:** 1 Ophthalmology, Edward Via College of Osteopathic Medicine, Monroe, USA; 2 Medical Education and Simulation, Illinois College of Osteopathic Medicine (IllinoisCOM) The Chicago School, Chicago, USA

**Keywords:** augmented reality (ar), education, ophthalmology, skills and simulation training, virtual reality (vr)

## Abstract

Virtual reality (VR) is a rapidly growing concept in the field of medical education, offering significant potential for innovation. Assessing how VR is used in ophthalmology to teach medical students, residents, and attendings will help identify areas where VR can be further developed. Moreover, analyzing the strengths and limitations of VR in medical education, surgical training, and patient education within ophthalmology will provide valuable insights into its impact on the field.

This scoping review aimed to (1) map the current applications of VR across ophthalmology education, (2) analyze the effectiveness of VR interventions for different learner groups, (3) identify gaps in current VR implementation, and (4) assess the quality of evidence supporting VR use in ophthalmology education.

A literature review was performed by scanning databases including PubMed, IEEE Xplore®, and Education Resources Information Center (ERIC). Relevant articles published from 2015 to 2023 were identified through the databases. Inclusion and exclusion criteria were formed for the articles to be further analyzed for eligibility. Articles were further categorized based on their VR application and target learners.

Most studies targeted the education of ophthalmology residents, highlighting a gap in VR use among students, other healthcare personnel, and patients. Future research should focus on expanding VR applications to these underrepresented groups. The rapid growth of VR in other areas of medical education suggests similar potential to enhance ophthalmology knowledge and skills across a more comprehensive audience. Research in the application of VR in ophthalmology education should extend beyond surgical training for residents to maximize its potential in improving patient care. For example, studies could explore how VR training improves the ophthalmic diagnostic skills of medical students, evaluate whether VR-based patient education enhances understanding of ophthalmic conditions and leads to improved patient outcomes, and whether VR training for opticians aids in the early detection of specific conditions like diabetic retinopathy. This review is limited by excluding grey literature, which may limit the material analyzed and overlook insights from non-peer-reviewed sources.

## Introduction and background

Virtual reality (VR) is an emerging tool in medical education, presenting exciting opportunities for improved learning experiences. Ophthalmology medical education faces notable challenges, including limited early exposure for medical students and significant variability in surgical training opportunities, which can hinder consistent skill development across trainees. Ophthalmology is a unique field where technical skills need to be developed to properly examine, diagnose, and treat various pathologies. Often in training, these technical skills are not fostered until students enter a clinical setting and practice on patients. Expanding VR in this field would allow medical students, residents, attendings, and other healthcare personnel to practice and improve their skill set prior to entering the clinical setting. Surgical training in the past consisted of wet lab training with cadaver eyes, grapes, and mannequins [1]. Ophthalmology surgery presents a specific challenge where microsurgery performed under a microscope leaves little room for trainees to assist and learn during surgical procedures. The expansion of VR in ophthalmology has led to the development of surgical simulators, such as the Eyesi® surgical simulator, which is increasingly used in residency training. This simulator offers cataract and vitreoretinal surgery modules, as well as basic skills training and complication management [2]. Beyond resident surgical training, VR shows promise in training and educating medical students. Most medical students do not get the experience and training of proper ophthalmologic physical examination, including the use of slit-lamp examination, dilated pupil exams with an indirect ophthalmoscope, and a direct ophthalmoscope. VR physical examination could help improve these technical skills before students enter a clinical setting.

Additionally, VR training could be beneficial to other healthcare personnel, like opticians, and help improve their dilated pupil examination and possibly improve diagnostic capabilities. Patients could benefit from VR education as well, helping to educate patients about their diagnosis and possibly improve adherence to treatment and follow-up. It could help improve healthcare literacy regarding ophthalmic conditions and empower patients to take a more active role in their healthcare. VR could be especially useful for high-risk patients, like those with diabetes or sickle cell, by illustrating visual disturbances that signal disease progression and when to seek care. Assessing how VR is used in ophthalmology to teach medical students, residents, and attendings will help identify areas where VR can be further developed. Moreover, analyzing the strengths and limitations of VR in medical education, surgical training, and patient education within ophthalmology will provide valuable insights into its impact on the field.

The primary goal of this study is to explore and outline the existing applications of VR within ophthalmology education and training. This objective will involve a comprehensive examination of the various ways in which VR has been integrated into the curriculum, including surgical training, diagnostic practice, and basic skills development. Another key objective of this study is to assess the effectiveness of VR interventions across different learner groups, including medical students, residents, and practicing ophthalmologists. This will help to understand how VR technology impacts learning outcomes and skill development for diverse experience levels. Furthermore, the literature review aims to pinpoint areas where the current use of VR in ophthalmology could be expanded, offering valuable insights into possible growth opportunities. These may include targeting different learner groups or exploring additional applications that could further enhance ophthalmology training. Lastly, this study aims to evaluate both the quality and quantity of evidence supporting the integration of VR into ophthalmology education, determining whether the current research is sufficient to justify widespread adoption in clinical or preclinical training programs.

## Review

Methods

**Figure 1:**
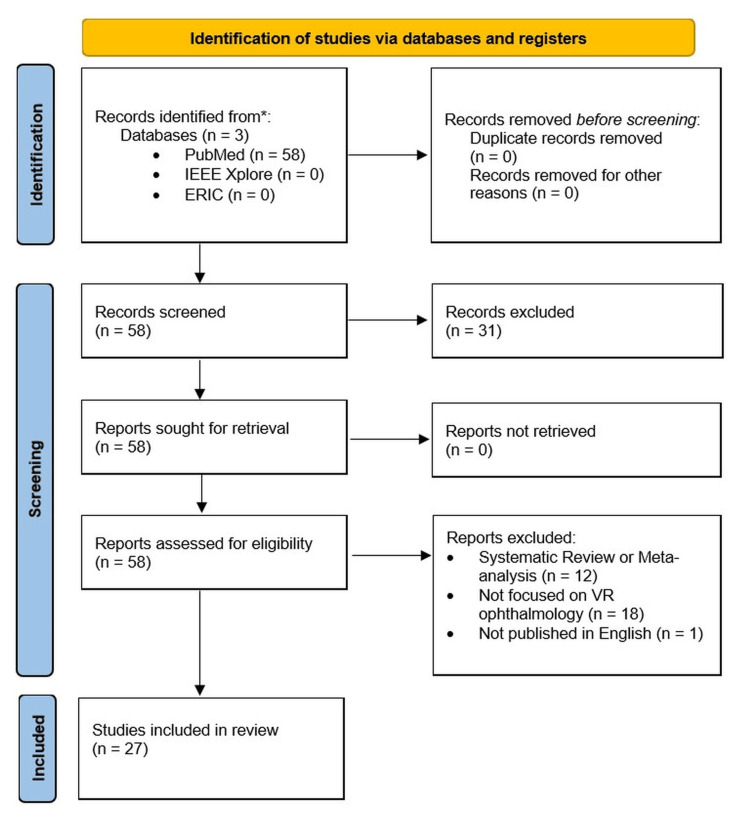
PRISMA-ScR flow diagram illustrating the methodology of screening articles PRISMA-ScR: Preferred Reporting Items for Systematic Reviews and Meta-Analyses extension for Scoping Reviews; VR: Virtual reality; ERIC: Education Resources Information Center

Literature review was performed following the Preferred Reporting Items for Systematic Reviews and Meta-Analyses extension for Scoping Reviews (PRISMA-ScR) guidelines. PRISMA-ScR is a set of guidelines used to help researchers systematically report their reviews. It ensures comprehensive and consistent presentations of findings, helping to improve reliability and quality in the review process [3]. These databases were scanned, including PubMed, IEEE Xplore®, and Education Resources Information Center (ERIC), using search terms "virtual reality" OR "VR" OR "augmented reality" AND "ophthalmology" AND "education" OR "training" OR "learning". Articles were filtered for publication dates from 2015 to 2023 to ensure findings remain relevant to current practices. Exclusion and inclusion criteria were developed. Studies were excluded if they were published outside of 2015-2023, if they were not available in English, if they did not focus on the education and training of medical students, residents, physicians, or patients, non-VR simulation studies, and systematic reviews and meta-analyses were excluded. Grey literature from non-peer-reviewed sources, including conference abstracts, newsletters, and technical reports, was excluded from the review. Two independent reviewers screened abstracts and titles for adherence to inclusion criteria and excluded studies that did not meet the criteria. Disagreements among the two independent reviewers were resolved through open discussion to reach a mutual agreement on the inclusion of a source. The methodology used to screen studies is illustrated in Figure 1.

The 27 included sources were reviewed further and categorized based on VR application type. The two independent reviewers reviewed each included article to determine which VR application category each study fit into. VR application type categories included surgical training, clinical applications in diagnosis and counseling, and physical examination education. Included sources were also grouped by their target learners. Target learner groups included medical students, residents, attending physicians, patients, and other healthcare personnel.

Results

The results from the 27 included sources were categorized into VR application type, depicted in Table 1. VR application types included surgical training and performance, clinical applications in diagnosis and counseling, and physical examination educational tools. Certain sources aligned with multiple categories and were therefore classified under each applicable category. Additionally, results were further categorized by target learners, including medical students, residents, attendings, and other healthcare personnel, as illustrated in Table 2. Table 3 demonstrates the categorization of included sources based on both the VR application type and the target learners. The predominant use of VR in ophthalmology was for surgical training and performance (n = 16). Physical examination education, including simulated pupil exams, slit-lamp training, and direct and indirect ophthalmoscopy, was the second most common use of VR training in ophthalmology (n = 8). The least studied application of VR in ophthalmology was in clinical diagnosis and counseling (n = 3). The most common use of VR in ophthalmology education was for training residents (n = 15). The use of VR among patients and other healthcare personnel was the least researched (n = 2, n = 2). The scoping review revealed that VR applications in ophthalmology have primarily centered on surgical training for residents, leaving significant gaps in other potential areas of implementation [4-30].

**Table 1:** Studies included in the review, organized by application OCT: Optical coherence tomography

VR Application Type	Examples	References
Surgical Training and Surgical Performance	Phacoemulsification Skill Development, Curvilinear Capsulorhexis Training, Manual Dexterity Evaluation, Virtual Vitreoretinal Surgery Programs, Inter-Procedural Skill Transfer	Bakshi et al., 2021 [4]; Ali Momin et al., 2022 [5]; Ng et al., 2018 [6]; Vergmann et al., 2017 [7]; Yen et al., 2017 [8]; Deuchler et al., 2022 [9]; Solyman et al., 2022 [10]; Vieira et al., 2020 [11]; Jacobsen et al., 2019 [12]; Zhang et al., 2023 [13]; Ferris et al., 2020 [14]; Wisse et al., 2017 [15]; Mathis et al., 2022 [16]; Thomsen et al., 2017 [17]; Sikder et al., 2015 [18]; Ng et al., 2023 [19]
Clinical Applications in Diagnosis, Counseling	Strabismus Diagnosis Training, Advanced Visualization of OCT Data, 3D Counseling Tools for Glaucoma, Interactive Patient Education, Simulation-Based Diagnostic Skill Enhancement	Moon et al., 2021 [20]; Maloca et al., 2018 [21]; Ramesh et al., 2024 [22]
Physical Examination Education	Simulated Pupil Examination, Simulation-Based Slit Lamp Training, Virtual Training for Direct and Indirect Ophthalmoscopy	Williams, 2022 [23]; Loidl et al., 2020 [24]; Gunasekeran et al., 2021 [25]; Deuchler et al., 2023 [26]; Boden et al., 2020 [27]; Deuchler et al., 2022 [28]; Rai et al., 2017 [29]; Tso et al., 2017 [30]

**Table 2:** Studies included in the review, grouped by target learners

Target Learners	References
Medical Students (n = 8)	Vergmann et al., 2017 [7]; Deuchler et al., 2022 [9]; Williams, 2022 [23]; Loidl et al., 2020 [24]; Deuchler et al., 2023 [26]; Boden et al., 2020 [27]; Deuchler et al., 2022 [28]; Tso et al., 2017 [30]
Residents (n = 15)	Momin et al., 2022 [5]; Ng et al., 2018 [6]; Vergmann et al., 2017 [7]; Yen et al., 2017 [8]; Vieira et al., 2020 [11]; Zhang et al., 2023 [13]; Ferris et al., 2020 [14]; Wisse et al., 2017 [15]; Mathis et al., 2022 [16]; Thomsen et al., 2017 [17]; Sikder et al., 2015 [18]; Ng et al., 2023 [19]; Moon et al., 2021 [20]; Maloca et al., 2018 [21]; Rai et al., 2017 [29]
Attending Physicians (n = 5)	Momin et al., 2022 [5]; Vergmann et al., 2017 [7]; Solyman et al., 2022 [10]; Jacobsen et al., 2019 [12]; Maloca et al., 2018 [21]
Patients (n = 2)	Ramesh et al., 2024 [22]; Gunasekeran et al., 2021 [25]
Other Healthcare Personnel (n = 2)	Momin et al., 2022 [5]; Maloca et al., 2018 [21]

**Table 3:** Classification of included studies by subject and learner population, displayed with reference numbers OCT: Optical coherence tomography

Subject/Learner Group	Patients	Medical Students	Residents	Attendings
Cataract Surgery		[9,10]	[4-6,8,12,14-19]	
Vitreoretinal Surgery			[7]	
Direct/Indirect Ophthalmoscopy		[27,28,30]	[29]	
Slit Lamp Training		[26]		
Manual Dexterity Assessment			[11,13]	
Pupil Examination		[23]		
OCT Visualization			[21]	[21]
Strabismus Diagnosis			[20]	
Patient Education/Counseling	[22,25]	[24]		

Discussion

The immersive and interactive nature of VR technology offers promising avenues for enhancing education, training, and patient care in ophthalmology beyond its current limited applications. Our analysis demonstrated that while surgical simulation shows positive outcomes for resident training, there remains untapped potential for VR in diagnostic training, patient education, and interdisciplinary collaboration. The concentration of VR applications in surgical training suggests that technological development has prioritized procedural skills over other competencies required in comprehensive ophthalmologic practice.

As presented in Table 3, the bulk of VR training applications in ophthalmology have been concentrated among residents rather than medical students and attending physicians. This distribution suggests a missed opportunity in developing further training modules for attending physicians, particularly on immersive techniques prior to their first clinical encounter with patients or complications.

Additionally, many of the VR applications currently being utilized for resident education could be readily adapted for medical students, as the platform for VR requires no additional hardware investment once the initial headset is acquired. Most contemporary VR software is device-agnostic, making implementation across different learner levels technically feasible. This adaptability presents an opportunity to broaden access to immersive ophthalmology training early in medical education, potentially enhancing foundational skills and interest in the specialty.

Manual dexterity assessment represents another area with significant untapped potential. This technology could extend beyond mere assessment to include targeted training techniques designed to improve dexterity, allowing trainees to identify their physical limitations and develop compensatory strategies prior to entering residency programs.

Pupil examination, which has only been studied in medical students according to our review, could be adapted at a more sophisticated level for residents. While medical students typically receive elementary training in pupil examination techniques, residents in ophthalmology must develop extensive expertise in this area throughout their careers. VR applications could enhance this training with more advanced and nuanced simulations that reflect the complexity encountered in clinical practice.

This study has several notable limitations, including a limited number of sources for inclusion and variability among VR systems, which complicates direct comparisons. The exclusion of grey literature may have narrowed the scope of analysis and missed significant perspectives from non-peer-reviewed sources, which can be especially relevant in the fast-paced development of VR technology. Second, publication bias may have influenced available literature, potentially overrepresenting positive outcomes while underreporting negative or neutral findings regarding VR applications. This may also explain the limited number of articles available for inclusion (n = 27); however, the limited number may also be further indications of the gap in the application of VR to the field.

Additionally, the heterogeneity of VR systems and implementation methods across studies made direct comparison challenging, potentially obscuring important effectiveness nuances across different contexts and user groups. The variability in VR hardware and software further limits the generalizability of the findings of this review. Many studies relied primarily on subjective satisfaction metrics rather than objective performance assessments, raising questions about the reliability of the reported benefits. The rapid advancement of VR technology means findings from older studies may not reflect current systems' capabilities. Finally, the predominance of single-institution studies with relatively small sample sizes limits generalizability across different training environments, suggesting caution when interpreting the broader applicability of findings to diverse ophthalmology education settings.

Future research should address the identified gaps in VR applications within ophthalmology. Studies should explore how VR training enhances the diagnostic capabilities of medical students and residents, particularly for conditions requiring spatial understanding and pattern recognition, such as retinal pathologies, where three-dimensional visualization could improve comprehension of complex structures. Research should evaluate whether VR-based patient education improves understanding, adherence, and clinical outcomes compared to traditional educational methods, potentially through randomized controlled trials that measure knowledge retention and behavioral changes following different educational interventions.

Additionally, investigations should determine if VR training among other healthcare personnel, such as opticians, technicians, and nurses, aids in the early detection of conditions like diabetic retinopathy, glaucoma, and age-related macular degeneration. VR training for other healthcare personnel could significantly reduce the burden on ophthalmologists while improving patient access to care. Moreover, VR could facilitate interdisciplinary collaboration between ophthalmologists, neurologists, and primary care providers, enhancing access to comprehensive care, supporting early disease detection, and contributing to better patient outcomes. The field would benefit from developing and validating standardized assessment metrics for VR-based training to enable more rigorous evaluation of educational outcomes, creating benchmarks that could be universally applied across training programs. Finally, longitudinal studies should be conducted to determine the long-term retention of skills acquired through VR training compared to conventional methods, tracking performance over months or years to establish whether VR offers sustainable advantages in skill development and maintenance.

## Conclusions

This review highlights the current emphasis on VR applications in ophthalmologic surgical training while identifying significant gaps across various user groups and clinical applications. The underrepresentation of VR use among medical students, attending physicians, patients, and allied healthcare personnel presents significant opportunities for technological development and implementation. These gaps highlight the need for more targeted efforts to integrate VR technology into all aspects of ophthalmology education and clinical practice, potentially transforming how these groups approach both training and patient care. VR technology could enhance the diagnostic capabilities of trainees in general ophthalmology, and its integration into subspecialties could ensure high-quality fellowship training. For example, VR simulations of ocular trauma could improve preparedness for real-life emergencies, especially in regions where ocular trauma is less common. Expanding VR applications in pediatric ophthalmology could improve the treatment of conditions such as strabismus. Moreover, VR could enhance refractive surgery by aiding surgical planning and simulating visual acuity with different lens powers, potentially increasing patient satisfaction post-cataract surgery.

Future research should expand beyond surgical simulation to include comprehensive clinical skills development, such as diagnostic abilities, physical examination techniques, and patient counseling. Immediate steps to implement VR in ophthalmology medical education include integrating VR models into the medical school curriculum, offering VR-based training at conferences, and educating clinicians on current VR applications in ophthalmologic diagnostics and patient education. By taking these initial steps, educators and clinicians can begin to harness the full potential of VR technology to improve both the training experience and patient care.
